# Clinical outcomes of influenza in individuals aged ≤20 years before and after COVID-19: a national analysis of mortality and complications

**DOI:** 10.1038/s41390-025-04759-7

**Published:** 2026-01-14

**Authors:** Bishes Khanal, Sandhya J. Kadam, Sagya Khanal, Fu-Sheng Chou, Ana C. Coll, Manish B. Malkar, Anita J. Moon-Grady, Laxmi V. Ghimire

**Affiliations:** 1 Kathmandu Medical College and Teaching Hospital, Kathmandu, Nepal; 2 Department of Pediatrics, Family HealthCare Network, Visalia, CA USA; 3 Nepal Medical College and Teaching Hospital, Kathmandu, Nepal; 4 Department of Neonatal-Perinatal Medicine, Kaiser Permanente Riverside Medical Center, Riverside, CA USA; 5 Division of Pediatric Cardiology, University of California, San Francisco, Fresno Regional Campus, Fresno, CA USA; 6 Division of Pediatric Cardiology, UCSF Benioff Children’s Hospital, Department of Pediatrics, University of California, San Francisco, San Francisco, CA USA

## Abstract

**Background:**

The COVID-19 pandemic has altered the typical annual pattern of influenza. We aim to assess its impact on influenza outcomes, especially on mortality, cardiovascular, and non-cardiovascular complications.

**Methods:**

This study utilized discharge records from the National Inpatient Sample (NIS) for 2019 and 2022. We analyzed the data of hospitalized influenza patients (20 years and younger) using descriptive statistics and logistic regression models.

**Results:**

29,270 influenza hospitalizations were identified in the pre-pandemic year and 29,860 in the post-mitigation year. The median age was 6 years (IQR: 2–12) in the post-mitigation year vs. 4 years (IQR: 1–9) in the pre-pandemic year, *P* < 0.001. Mortality and major cardiovascular complications, including myocarditis and tachyarrhythmias, showed no differences. Respiratory failure was more common in post-mitigation (21.4% vs. 12.7%), with nearly twice the risk in adjusted models [aOR = 1.92 (1.68–2.19), *P* < 0.001]. Invasive mechanical ventilation use decreased in the post-mitigation year by 30% [aOR = 0.7 (0.55–0.89), *P* = 0.004].

**Conclusion:**

Mortality and cardiovascular complications remained stable in pre- and post-mitigation year; however, post-pandemic influenza showed an increased risk of respiratory failure with a decreased invasive mechanical ventilation use. This suggests a shift in disease presentation and clinical management strategies in the post-mitigation period.

**Impact:**

- Influenza’s clinical profile in children has changed post-COVID, with increased respiratory failure and a shift to older affected age groups, while mortality and severe cardiovascular complications remain stable.
- This study is the first to systematically compare pediatric influenza outcomes, including all major complications and mortality, pre- and post-COVID-19, revealing specific shifts in disease presentation and demographics.
- Informs clinical vigilance, diagnostic strategies, and public health planning for pediatric influenza by highlighting shifts in hospitalization patterns and complications observed during the post-mitigation period.
- Findings support preparedness efforts recognizing temporal disruptions surrounding the pandemic.

## Introduction

Influenza is mainly a respiratory illness caused by Influenza A and B viruses. It occurs as seasonal outbreaks, usually during colder months, as an epidemic. Strains of the Influenza A virus of zoonotic origin involve the outbreaks of sporadic pandemics.^1^

Not all patients suffering from the Influenza virus are seen by medical professionals, and not all get tested for influenza. So it is impossible to identify them all through the surveillance systems. Therefore, mathematical models are used to study the impact of the influenza virus.^2^

Influenza remains a significant global health problem. The World Health Organization (WHO) estimates approximately 1 billion infections, 3 to 5 million cases of seasonal illness, and between 290,000 to 650,000 respiratory deaths worldwide each year.^1,3^ In the United States, the Centers for Disease Control and Prevention (CDC) reports that influenza has caused 9.3 million–41 million illnesses, 120,000–710,000 hospitalizations, and 6,300–52,000 deaths annually in the United States in the last fifteen years.^4^

Before the COVID-19 pandemic, annual influenza infection had predictable surges and outcomes, including rare complications such as pneumonia and myocarditis. In 2020, the emergence of COVID-19 and mitigation strategies disrupted the usual pattern of influenza, reducing global influenza circulation during 2020–2021.^5,6^ After easing the mitigation measures in 2022, influenza resurged, with concerns that a lack of recent exposure might have made the influenza illness more serious.^5,7^ During the COVID-19 pandemic, isolation measures and mitigation efforts taken to slow the spread of SARS-CoV-2 had indirect effects on the dynamics of outbreaks of other seasonal viruses, especially respiratory syncytial virus (RSV) and influenza.^8^

Clinical presentation, hospitalization rates, influenza-related complications, and the number of deaths also showed changes during the post-mitigation period compared to the pre-pandemic period. A significant decrease in influenza A cases was noted during the COVID-19 pandemic, and hospitalized children were found to have more severe symptoms afterward.^9^

To our knowledge, no study has systematically compared the impact of COVID-19 on influenza presentation and complications, including mortality rates and cardiovascular and non-cardiovascular complications. This study aims to compare in-hospital mortality and major cardiovascular complications among hospitalized children and young adults with influenza infection during the pre-pandemic and post-mitigation periods.

## Methods

This retrospective study utilized hospitalized discharge records from the National Inpatient Sample (NIS) for 2019 and 2022. The NIS is the largest publicly available all-payer inpatient healthcare database in the United States. It is a part of a comprehensive set of databases and analytical tools designed for the Healthcare and Utilization Project (HCUP), sponsored by the Agency for Healthcare Research and Quality (AHRQ). The NIS sampling frames include data from all the states participating in HCUP, covering more than 97 percent of the US population. It is a deidentified database approximating a stratified 20% sample of discharges from U.S. community hospitals, excluding long-term acute care hospitals and rehabilitation facilities. An approach of excluding unique identifiers for the state and hospitals in the newer version has ensured patient confidentiality. A detailed description of the database can be accessed through the HCUP website.^10^

Our study’s inclusion criteria include individuals less than or equal to 20 years old who were hospitalized with the diagnosis of influenza. The age limit of ≤ 20 years was chosen to include all pediatric and adolescent hospitalizations as defined by the NIS and to align with prior pediatric outcomes studies using the same database.

**Fig. 1:**
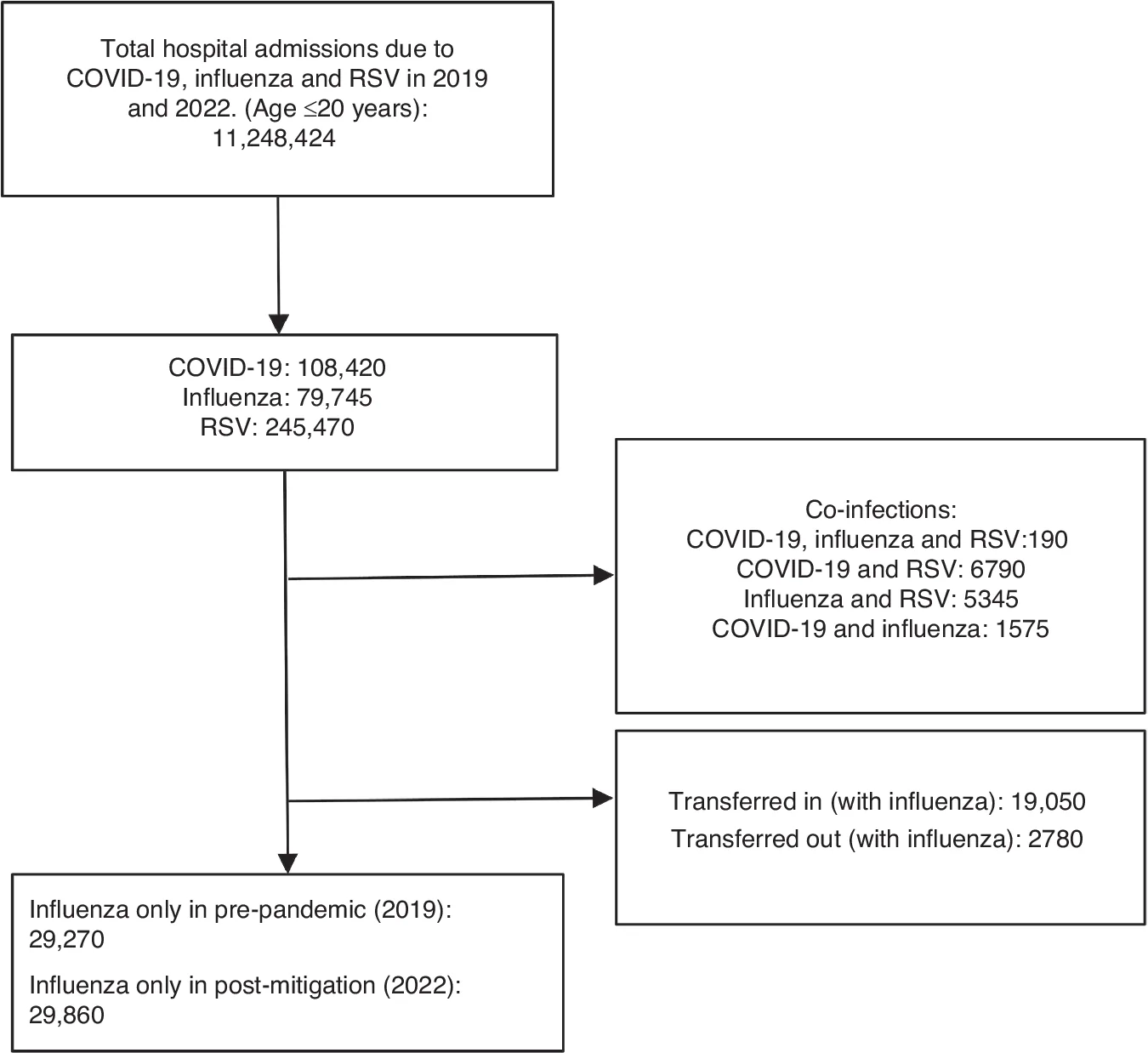
Flow diagram for in-hospital admissions.

The International Classification of Diseases, tenth revision, Clinical Modification (ICD-10-CM) was employed to identify the cases of influenza and other variables studied. To reduce confounding effects and strengthen internal validity, we excluded individuals with co-respiratory infections and individuals who were transferred in and out of the hospital. Co-infections were identified using ICD-10 codes for RSV and SARS-CoV-2. Transfer-in and transfer-out admissions were excluded to prevent duplicate counts and ensure complete outcome data. Further, we omitted admissions that did not have complete data for analysis (Fig. 1).

To identify the cases of influenza within the database, we utilized the following ICD-10 codes: J10. xx to J11.xx, J09X, J09X1, J09X2, J09X3 and J09X9. This study aims to assess the influence of the COVID-19 pandemic on influenza-associated complications. A comparative analysis was carried out to evaluate the temporal and spatial changes in influenza, centered on analyzing the shifts in mortality trends and cardiovascular-related complications. To observe these changes, 2019 was considered the ‘pre-pandemic period’, representing the last full year before pandemic-related disruptions, whereas 2022 was considered the ‘post-mitigation period’, reflecting the year when healthcare systems started adapting to the long-term effects of COVID-19. The years 2020 and 2021 were excluded because widespread COVID-19 mitigation measures resulted in historically low influenza circulation and inconsistent hospitalization patterns, making those years unsuitable for temporal comparison.

As mentioned, the study’s primary outcome of interest includes mortality and cardiovascular complications. Major cardiovascular complications comprise myocarditis, tachyarrhythmias, sudden cardiac arrest, and heart block. Secondary outcomes encompass non-cardiovascular complications, including respiratory failure, acute respiratory distress syndrome, extracorporeal membrane oxygenation, invasive mechanical ventilation, non-invasive mechanical ventilation, acute renal failure, dialysis, cardiopulmonary resuscitation (CPR), and length of hospital stay. A detailed account of the codes used for individual variables can be accessed in Supplementary Table S1.

A rigorous statistical analysis was performed, including descriptive and inferential statistics, followed by univariable and multivariable logistic regression models. We implemented clustering, stratification, and weighting following the recommendations set forth by HCUP to produce national estimates and maintain the precision of the statistical analysis. All analyses incorporated NIS survey design elements (discharge weights, hospital strata, and cluster variables) using Stata’s svy and R’s survey packages to ensure nationally representative estimates. Continuous variables, such as age and length of stay, were reported as medians and interquartile ranges. Categorical variables were analyzed using weight-adjusted chi-square tests. Neighborhood ZIP Codes classify the estimated median household income of residents in a patient’s ZIP Code into four quartiles. The quartiles are identified from lowest to highest, indicating the lowest-income neighborhoods to the highest-income neighborhoods.

For regression modeling, univariable analysis was undertaken, followed by multiple logistic regression models after accounting for the additional covariates with consistent and reliable ICD-10 codes, including age, sex, prematurity, obesity, diabetes, congenital heart disease, chromosomal anomalies, ZIP code of household neighborhood, and disease severity. Analyses were performed using Stata 15.1 and R version 4.3 with RStudio (version 1.2). R packages included tidyverse, survey, ggplot2, and tableone.^11^

## Results

After accounting for the inclusion and exclusion criteria, our descriptive analyses revealed 29,270 hospitalized influenza cases in the pre-pandemic year and 29,860 in the post-mitigation year. Among these, 13,205 (45.1%) were females in the pre-pandemic year, whereas 13,795 (46.2%) were females in the post-mitigation year.

Age, a variable in our analysis, revealed intriguing findings. A trend indicating older age groups [6 years (IQR: 2–12)] being more affected in the year after, compared to the year before the outbreak [4 years (IQR: 1–9)], *P* < 0.001 was observed (Table 1). To better understand this trend, we stratified age groups into four categories. All three age groups, encompassing 0–2 years, 3–5 years, and 6–10 years, were more affected in the pre-pandemic years, whereas the older age group of 11–20 years was more affected in the post-mitigation years. We further noticed that children predominantly within the age group of 3–5 years were hospitalized with influenza in both pre- and post-outbreak years (Table 1).

**Table 1:** Influenza complications before and after pandemic (Pre-pandemic period: 2019 and post mitigation period: 2022). Numbers in the parentheses are the percentages.

	Pre pandemic	Post mitigation	*P*-value
Total number	29270	29860	
Female	13205 (45.1)	13795 (46.2)	0.246
Mortality	70 (0.2)	80 (0.3)	0.752
Age (median [IQR])	4 (1–9)	6 (2–12)	< 0.001
Age group			< 0.001
0–2	5075 (17.3)	3645 (12.2)	
3–5	11,575 (39.5)	11,230 (37.6)	
6–10	6360 (21.7)	6270 (21.0)	
11–20	6260 (21.4)	8715 (29.2)	
Zip code*			0.182
1st quartile	10,220 (35.3)	9875 (33.3)	
2nd quartile	7010 (24.2)	7035 (23.7)	
3rd quartile	6940 (24)	7025 (23.7)	
4th quartile	4750 (16.4)	5730 (19.3)	
CHD^a^	1010 (3.5)	915 (3.1)	0.292
Asthma	7075 (24.2)	7785 (26.1)	0.03
Chromosomal anomalies	720 (2.5)	720 (2.4)	0.868
Prematurity	405 (1.4)	360 (1.2)	0.467
Obesity	615 (2.1)	955 (3.2)	0.001
Diabetes	730 (2.5)	1015 (3.4)	0.004
**Cardiovascular complications**			
Myocarditis	45 (0.2)	65 (0.2)	0.414
Tachyarrhythmia	235 (0.8)	350 (1.2)	0.05
Heart block	190 (0.6)	170 (0.6)	0.589
Sudden cardiac arrest	55 (0.2)	70 (0.2)	0.568
**Non-cardiovascular complications**			
Respiratory failure	3730 (12.7)	6380 (21.4)	<0.001
Acute respiratory distress syndrome	100 (0.3)	100 (0.3)	0.950
ECMO^b^	25 (0.1)	40 (0.1)	0.413
Invasive mechanical ventilation(IMV)	930 (3.2)	870 (2.9)	0.443
Non-invasive mechanical ventilation (NIMV)	845 (2.9)	965 (3.2)	0.486
Acute renal failure	900 (3.1)	1275 (4.3)	0.003
Dialysis	30 (0.1)	65 (0.2)	0.160
CPR (cardiopulmonary resuscitation)	60 (0.2)	20 (0.1)	0.046
LOS (median[IQR]) in days	2 (1–4)	2 (1–4)	0.953

^a^Congenital Heart Disease.

^b^Extracorporeal membrane oxygenation.

*Zip Code: Neighborhood ZIP Codes classify the estimated median household income of residents in a patient’s ZIP Code into four quartiles. The quartiles are identified from lowest to highest, indicating the lowest-income neighborhoods to highest-income neighborhoods.

We studied the prevalence of associated chronic medical conditions in children hospitalized for influenza. Among such co-morbid conditions, children and young adults with a history of asthma/airway disease were more frequent in the post-mitigation period (26%, *n* = 7,785) compared to the pre-pandemic period (24.2%, *n* = 7,075), *p* = 0.03. A higher prevalence of diabetes was exhibited in the patients hospitalized in the post-pandemic year (3.4%, *n* = 1,015) compared to the pre-pandemic (2.5%, *n* = 730), *P* = 0.04. Obesity was more evident among children admitted following the pandemic (3.2%, *n* = 955) than those admitted before the pandemic (2.5%, *n* = 615), *P* = 0.001. Details of patient baseline characteristics and prevalence of comorbid conditions are represented in Table 1.

Mortality and major cardiovascular complications were similar before and after the pandemic in descriptive analysis and regression models. The proportion of mortality was (0.2%, *n* = 70) and (0.3%, *n* = 80) in the pre-pandemic and post-mitigation years, respectively, *P* = 0.752. After adjusting for confounders, the adjusted odds ratio (aOR) for mortality was [aOR = 0.94 (0.44–1.99), *P* = 0.88]. In this model, obesity was associated with a 7.7-fold increase in the odds of mortality, while diabetes was linked to a fourfold increase. Similarly, adjusted odds ratio (aOR) for myocarditis was [aOR = 1.14 (0.49–2.63), *P* = 0.75], for heart block [aOR = 0.70 (0.44–1.13), *P* = 0.15], for sudden cardiac arrest [aOR=1.07 (0.46–2.46), *P* = 0.87], and tachyarrhythmias [aOR = 1.12 (0.87–1.85), *P* = 0.20] (Table 2).

**Table 2:** Regression model analysis, outcomes in post-mitigation period compared to pre-pandemic period.

Outcome variables	Unadjusted model		Adjusted model^*^	
	OR (95% CI)	*P*-Value	OR (95% CI)	*P*-value
Mortality	1.12 (0.54–2.29)	0.756	0.94 (0.44–1.99)	0.879
**Cardiovascular complications**				
Tachyarrhythmia	1.46 (1.01–2.12)	0.044	1.2 (0.87–1.85)	0.199
Heart block	0.87 (0.55–1.39)	0.577	0.70 (0.44–1.13)	0.148
Sudden cardiac arrest	1.24 (0.56–2.75)	0.583	1.07 (0.46–2.46)	0.869
Myocarditis	1.41 (0.6–3.31)	0.422	1.14 (0.49–2.63)	0.754
**Non-cardiovascular complications**				
Respiratory failure	1.8 (1.68–2.05)	< 0.001	1.92 (1.68–2.19)	< 0.001
Acute renal failure	1.4 (1.15–1.7)	0.001	1.14 (0.92–1.40)	0.214
Invasive mechanical ventilation(IMV)	0.91 (0.74–1.12)	0.404	0.7 (0.55–0.89)	0.004
Non-invasive mechanical ventilation(NIMV)	1.12 (0.91–1.38)	0.276	0.9 (0.72–1.12)	0.361
ECMO^a^	1.56 (0.51–4.79)	0.430	1.31 (0.43–3.98)	0.628

^a^Extracorporeal membrane oxygenation.

^*^Adjusted for age, sex, prematurity, obesity, diabetes, congenital heart disease, chromosomal anomalies, ZIP code of household neighborhood, and disease severity.

Significant differences emerged in several secondary, non-cardiovascular outcomes. Respiratory failure was more prevalent in the post-mitigation year (21.4%, *n* = 6,830) compared to the pre-pandemic period (12.7%, *n* = 3,730), *P* < 0.001. An unadjusted logistic regression model revealed a 1.8-fold higher risk of respiratory failure in a post-mitigation year compared to the pre-pandemic year [aOR = 1.8 (1.68–2.05), *P* < 0.001]. In multiple logistic regression models, after adjusting for potential confounders, the risk of respiratory failure was nearly twice as high in the subsequent year compared to the year before the outbreak [aOR = 1.92 (1.68–2.19), *P* < 0.001] (Table 2). Although the use rate of invasive mechanical ventilation (IMV) seemed similar in pre-pandemic and post-mitigation hospitalization in the descriptive analysis, a contrasting observation was found in the multiple logistic regression analysis. After adjusting for covariates, mathematical modeling indicated a 30% drop in the use of IMV during the post-mitigation year compared to the pre-pandemic one [aOR = 0.7 (0.55–0.89), *P* = 0.004]. Acute respiratory distress syndrome, extracorporeal membrane oxygenation (ECMO), non-invasive mechanical ventilation, and cardiopulmonary resuscitation showed no significant differences in regression models.

In descriptive analysis, acute renal failure seemed higher during post-mitigation years; the difference in the rates was statistically insignificant in regression models. The need for dialysis was similar in pre- pandemic and post-mitigation year. Further, the patients admitted for influenza in both years had similar lengths of hospital stay (Table 1).

## Discussion

Using the National Inpatient Sample (NIS) for 2019 and 2022, we report the following major findings: (1) The in-hospital mortality and cardiovascular complications were similar before and after the pandemic. (2) The risk of respiratory failure increased in 2022, yet the use of invasive mechanical ventilation declined over the same period. (3) Older children experienced a greater adverse impact in the year following the outbreak compared to the year before.

Our analysis revealed similar rates of mortality in the pre-pandemic and post-mitigation years, a trend consistent with the figures reported by the CDC and corroborated by existing literature. As per the CDC report, the 2022–23 influenza season had high severity in children and young adolescents (5-17 years) compared with previous seasons in influenza-associated outpatient visits, hospitalization rates, and deaths.^12^ The increased disease burden led to a large volume of infections post-pandemic. Therefore, the absolute number of influenza-related deaths appeared higher. However, the overall mortality rate per infection remained relatively stable at 0.5 per 100,000 population among individuals aged 5–17 years.^12,13^ In line with our findings, a retrospective study by Gentile et al. in their multicenter, cross-sectional study also reported no significant difference in the case fatality rates during pre-pandemic and post-mitigation periods.^14^

The higher rates of obesity and diabetes was observed in 2022. It may reflect broader lifestyle and metabolic changes during the pandemic period, including reduced physical activity and dietary shifts. These comorbidities are known independent predictors of severe respiratory outcomes and were also associated with higher mortality in our adjusted models, underscoring their clinical importance.

Influenza generally has a lower risk of developing cardiovascular complications than other respiratory viruses, including COVID-19 and RSV.^15,16^ Before 2020, cardiovascular complications were recognized but uncommon – only a handful of pediatric influenza cases each year would progress to fulminant myocarditis or cardiac failure.^17^ During the pandemic, practically no influenza myocarditis in children was reported during 2020–21, because there were almost no pediatric flu infections. Similarly, influenza myocarditis remains rare in post-COVID years, with only a few studies reporting such cases.^18,19^ Owing to the lower cardiovascular virulence of the virus and the rarity of such outcomes, our study showed no significant difference in cardiovascular complications in the pre-pandemic and post-mitigation period.

Respiratory failure was observed to increase in the post-mitigation period. Influenza is known to cause pediatric respiratory complications ranging from pneumonia to acute respiratory distress syndrome (ARDS) and respiratory failure.^20^ Studies have reported that, after the pandemic, children with influenza exhibited more respiratory symptoms, including cough, sore throat, and nasal congestion, culminating in more severe clinical courses.^9^ This observation could primarily be attributed to epidemiologic rebound phenomena following relaxation of mitigation measures and increased susceptibility. During the COVID-19 pandemic, broad use of nonpharmacological interventions such as hand washing, masking, and physical distancing greatly reduced the circulation of many respiratory viruses. As a result, exposure to seasonal pathogens like influenza declined for an extended period. When these measures were later relaxed, a larger proportion of children and adolescents had not encountered these viruses in the preceding seasons, which created a temporary increase in the number of individuals who were susceptible to infection. This population-level pattern, described in recent reports, helps explain the higher burden of respiratory complications seen when influenza activity returned, without suggesting any change in immune function or in the behavior of the virus. Following the relaxation of mitigation efforts and NPIs, these viruses re-emerged, resulting in more severe infections, manifested in part by increased incidence of respiratory failure.^21^ In further exploring the underlying cause of these changes in complications before and after the pandemic, it is also important to consider potential alterations in the virulence of respiratory viruses during these times.^22^ Regardless of higher prevalence of respiratory failure, hospital length of stay remained unchanged, possibly reflecting improved early recognition and standardized respiratory management protocols developed during the pandemic.

A decreased risk of invasive mechanical ventilation use was noted, despite such rise in respiratory failure. Increased healthcare costs, shortage of protective equipment, and low numbers of ICU beds and ventilators revealed significant gaps in patient care during COVID-19.^23^ In an aim to conserve ventilator resources and reduce the risks associated with IMV, alternatives to ventilator support were considered in the form of NIMVs.^24^ Post-mitigation, these non-invasive strategies have remained prevalent in treating respiratory failure, potentially leading to reduced IMV usage despite severe cases. However, our results do not indicate the increased use of NIMVs. The absence of significant changes in NIMV use along with ARDS, ECMO, and CPR suggests that the overall critical care burden for severe influenza remained relatively stable between periods. These null findings also highlight the limitations of administrative data in capturing nuanced ICU practices or coding variability.

A shift in age distribution was reported during and after the pandemic. In line with our findings, three studies reported increasing influenza infections in older children in the post-mitigation period. A study based in Argentina by Gentile et.al. found that post-mitigation cases involved older children.^14^ Similarly, a multicentre, cross-sectional study based in Saudi Arabia stated that patients infected with the influenza virus between 2020 and 2021 were older than patients infected before the COVID-19 pandemic.^25^ Likewise, a study based in China by Jia et al. observed increased influenza infection in older children in the post-mitigation period as compared to newborns and young children in the pre-pandemic period.^26^ Increased influenza cases in the older cohort could be attributed to school attendance, leading to higher exposure across these age groups. Such similar observations over varied demographics enhance the validity and reliability of our findings.

Although adjusted models showed no significant difference in renal outcomes, the observed trend toward higher acute renal failure rates post-pandemic warrants further investigation, as influenza-associated acute kidney injury is a recognized contributor to morbidity in hospitalized children.

The main strength of our study lies in using a nationwide database (NIS) based on population sampling, providing us with a large sample size. Furthermore, a robust methodology and rigorous analysis enhance the credibility of our findings. However, there are constraints with this approach. NIS includes inpatient records only, missing out on the outpatient database, limiting the applicability of our findings in such settings. Exclusion of the less severe cases managed at the outpatient department could shift the data to more severe cases, potentially overestimating the severity of the diseases. Additionally, the reliance on the ICD-10 codes for diagnosing respiratory infections could be a limitation, as these codes may not provide detailed information on the diagnostic procedures and confirmatory tests used. The presence of errors or data omissions in the NIS, as with many medical databases, can contribute to potential inaccuracies and bias. As this database is encounter-based, we excluded children transferred in or out to prevent double-counting. This approach may omit some patients from the analysis and thus influence our outcomes, a limitation we explicitly acknowledge in the methods. This may underestimate disease severity, as most severe ones are the ones who are transferred.

In conclusion, our findings suggest that the clinical profile of influenza in pediatric populations has shifted in the aftermath of the COVID-19 pandemic. While mortality and cardiovascular complication rates remained stable, there was a significant increase in respiratory complications and a notable shift in the age distribution of affected children. These trends point to a potential transformation in influenza disease dynamics, with implications for clinical vigilance, diagnostic strategies, population-level prevention, vaccination recommendations, and post-COVID-19 mitigation preparedness in pediatric care.

## Supplementary information

Supplemental Table S1

## Data Availability

The datasets generated during and/or analysed during the current study are available in the HUCP repository, and can be accessed through http://www.hcup-us.ahrq.gov/nisoverview.jsp.
