# Supplementary material for: Clinical outcomes of influenza in individuals aged ≤20 years before and after COVID-19: a national analysis of mortality and complications
Source: Pediatr Res. 2026 Jan 14;100(3):1353–8. doi: 10.1038/s41390-025-04759-7 (PMC13672086; doi:10.1038/s41390-025-04759-7)
Supplement: Supplementary file 1 [file 41390_2025_4759_MOESM1_ESM.pdf]

Supplemental Table S1. Variables and the corresponding ICD-10 codes.

**Variables**	**ICD-10 code**
**Extracorporeal membrane oxygenation**	5A1522F, 5A1522G, 5A1522H
**Invasive mechanical ventilation(IMV)**	0DH57BZ, 0DH58BZ, 0BH17EZ, 0BH18EZ, 0B717DZ, 0B718DZ, 0BH07DZ, 0WHQ7YZ, 5A1935Z, 5A1945Z, 5A1955Z
**Non-invasive mechanical ventilation(NIMV)**	5A09357, 5A09457, 5A09557, 5A09358, 5A09458, 5A09558
**Influenza**	J10.xx to J11.xx, J09X, J09X1, J09X2, J09X3, J09X9
**Congenital heart disease**	Q20.xx to Q26.xx
**Diabetes**	E08.xx to E13.xx
**Obesity**	E65, E66.xx
**Myocarditis**	I51.4, I40.8, I40.9, B33.20, I40.1, I40.0, J10.82, I41, B33.22, I40
**Tachyarrhythmias**	I47.1, I47.2, I49.01, I49.0, I48.0, I48.3, I48.4
**Heart block**	I44.1, I44.2
**Sudden cardiac arrest**	I46.x
**Asthma/reactive airway disease**	J45.xx
**Prematurity (<37 weeks of gestation)**	P072.1 to P072.6, P073.x
**Chromosomal anomalies**	Q90.xx-Q99.xx
**Acute renal failure**	N17, N170, N171, N172, N178, N179
**Acute respiratory failure**	J9600, J9601, J9602
**Dialysis**	5A1D00Z, 5A1D60Z, 3E1M39Z, 5A1D70Z, 5A1D80Z
**CPR (cardiopulmonary resuscitation)**	5A12012
